# Global ecological regionalization of 15 *Illicium* species: nature sources of shikimic acid

**DOI:** 10.1186/s13020-018-0186-9

**Published:** 2018-06-15

**Authors:** Xiang Zhang, Xiangxiao Meng, Jie Wu, Linfang Huang, Shilin Chen

**Affiliations:** 1 Institute of Medicinal Plant Development, Chinese Academy of Medical Sciences and Peking Union Medical College, Beijing, 100193 China; 2 Institute of Chinese Materia Medica, China Academy of Chinese Medical Sciences, Beijing, 100700 China

**Keywords:** Shikimic acid, *Illicium* plants, Geographic information system for global medicinal plants, Ecological suitable areas

## Abstract

**Background:**

*Illicium* plants are relevant officinal and ornamental species that are native in Eastern Asia, and they are the main sources of shikimic acid. Shikimic acid is an important component of Tamiflu, which is recognized for its ability to resist avian influenza by the World Health Organization. To determine areas where 15 *Illicium* species can be grown and to understand the importance of species diversity, we should enhance the prediction of suitable areas.

**Methods:**

In this study, the global potential distribution of 15 *Illicium* species was predicted using a geographic information system for global medicinal plants.

**Results:**

Results showed that the possible suitable areas for these plants in China covered 1357.68 × 10^4^ km^2^ (56%), and the second-largest area spanning 527.42 × 10^4^ km^2^ was found in the United States. *Illicium verum* Hook, an edible species with the highest shikimic acid content among them, grew in areas of 59.92 × 10^4^ (48%), 64.04 × 10^4^ (19%), and 60.53 × 10^4^ km^2^(18%) in China, the United States, and Brazil, respectively. *Illicium.difengpi* B. N. Chamg, an endangered species, was distributed in an area of 19.03 × 10^4^ km^2^ or 95% of the total area in China.

**Conclusions:**

This research provided a guarantee for the demand of Tamiflu, presented strategies that helped protect endangered species, and provided a reference for species cultivation and introduction.

**Electronic supplementary material:**

The online version of this article (10.1186/s13020-018-0186-9) contains supplementary material, which is available to authorized users.

## Background

Shikimic acid (3,4,5-trihydroxy-1-cyclohexene-1-carboxylic acid) is commonly known as the main natural raw material of Tamiflu, which is recognized as the first drug to treat avian influenza in clinical settings [1–3]. The main sources of shikimic acid are *Illicium* plants, which are naturally distributed in Southeast China, the United States, Burma, and Vietnam [4] (Table 1). A total of 34 kinds of *Illicium* plants exist, and 15 of them (*I. verum* Hook. f., *I. henryi* Diels, *I. majus* Hook. f. & Thoms., *I. simonsii* Maxim., *I. micranthum* Dunn, *I. dunnianum* Tutch., *I. lanceolatum* A. C. Smith, *I. fargesii* Finet & Gagnep, *I. jiadifengpi* B. N. Chang, *I. difengpi* B. N. Chamg, *I. ternstroemioides* A. C. Smith, *I. macranthum* A. C. Smit, *I. oligandrum* Merr, et al. Chun, *I. brevistylum* A. C. Smith, and *I. pachyphyllum* A. C. Smith) can be used as Chinese herbal medicines for the treatment of rheumatoid arthritis, injuries, abdominal distention, and vomiting [5, 6]. Currently, they are mainly distributed in South and Southeast China, such as Hunan, Fujian and Guangxi. Among these species, *I. verum* Hook. f., an edible plant which is listed in the Pharmacopoeia of China (2015 edition) and European Pharmacopoeia (7th edition). *I. difengpi* B. N. Chamg is listed in the Pharmacopoeia of China (2015 edition) and recorded in the Information System of Chinese Rare and Endangered Plants (http://rep.iplant.cn/protlist).

**Table 1:** Distribution points of 15 kinds of *Illicium* plants

Latin names of species	Distribution	Sampling points
*I. verum*	In China, western and southern of Guangxi, southern of Zhejiang, Jiangxi, Hunan and Chongqing, south-central Fujian, southwest of Guangdong and Guizhou, southeast of Hainan, Yunnan, etc.	61
*I. henryi*	In China, Anhui, Jiangxi, Fujian, Henan, Guangdong, Guangxi, Chongqing, Yunnan, southern of Shanxi and Gansu, northern of Hubei, from west to northwest of Hubei, from east to southeast of Sichuan, from eastern to northern of Guizhou, etc.	171
*I. majus*	Guangxi, Guizhou, Chongqing, Yunnan, Hunan, southwest of Hubei, from western to northern of Guangdong, from central to south central of Sichuan in China, southern of Burma, northern of Vietnam, etc.	128
*I. simonsii*	Southwest of Sichuan (Xichang, Huili, Puge),from northwest to southeast of Guizhou, northwest, northeast and central Yunnan in China, northern of Burma, northeast of India, etc.	95
*I. micranthum*	In China, Hunan, Guangdong, Hongkong, Sichuan, Guizhou, Chongqing, Yunnan, western of Hubei, from central to northern of Guangxi, etc.	93
*I. dunnianum*	In China, Guangdong, Hongkong, Guangxi, southern of Fujian, southwest of Hunan, southern and southwest of Guizhou, southeast of Sichuan, etc.	80
*I. lanceolatum*	In China, Anhui, Zhejiang, Jiangxi, Fujian, Hubei, Hunan, Guizhou, southern of Jiangsu, southeast of Henan, etc.	76
*I. fargesii*	In China, Chongqing, western of Hubei, northeast of Yunnan, from northwest to southwest of Hunan, from northern to northeast of Guangxi, from eastern to northeast of Guizhou, from eastern to central of Sichuan, etc.	72
*I. jiadifengpi*	In China, Jiangxi, Fujian, Hongkong, southern of Anhui, southwest of Zhejiang, northeast of Guangxin, northern of Guangdong, southeast of Hubei, from southern to eastern of Hunan, etc.	69
*I. difengpi*	In China, Guangxin, southeast of Yunnan, etc.	61
*I. ternstroemioides*	In China, Fujian, Guangdong, Hongkong, Guangxi, southern of Hunan, southeast of Yunnan, etc.	58
*I. macranthum*	In China, southeast and western of Yunnan, southeast of Tibet, etc.	42
*I. oligandrum*	South of Guangxi, Hainan in China, Vietnam, etc.	37
*I. brevistylum*	In China, Guangdong, Guangxi, southern of Hunan, southeast of Yunnan, Fujian, Hongkong, etc.	29
*I. pachyphyllum*	In China, southwest of Guangdong and Hunan, southeast and northeast of Guizhou, etc.	22

**Fig. 1:**
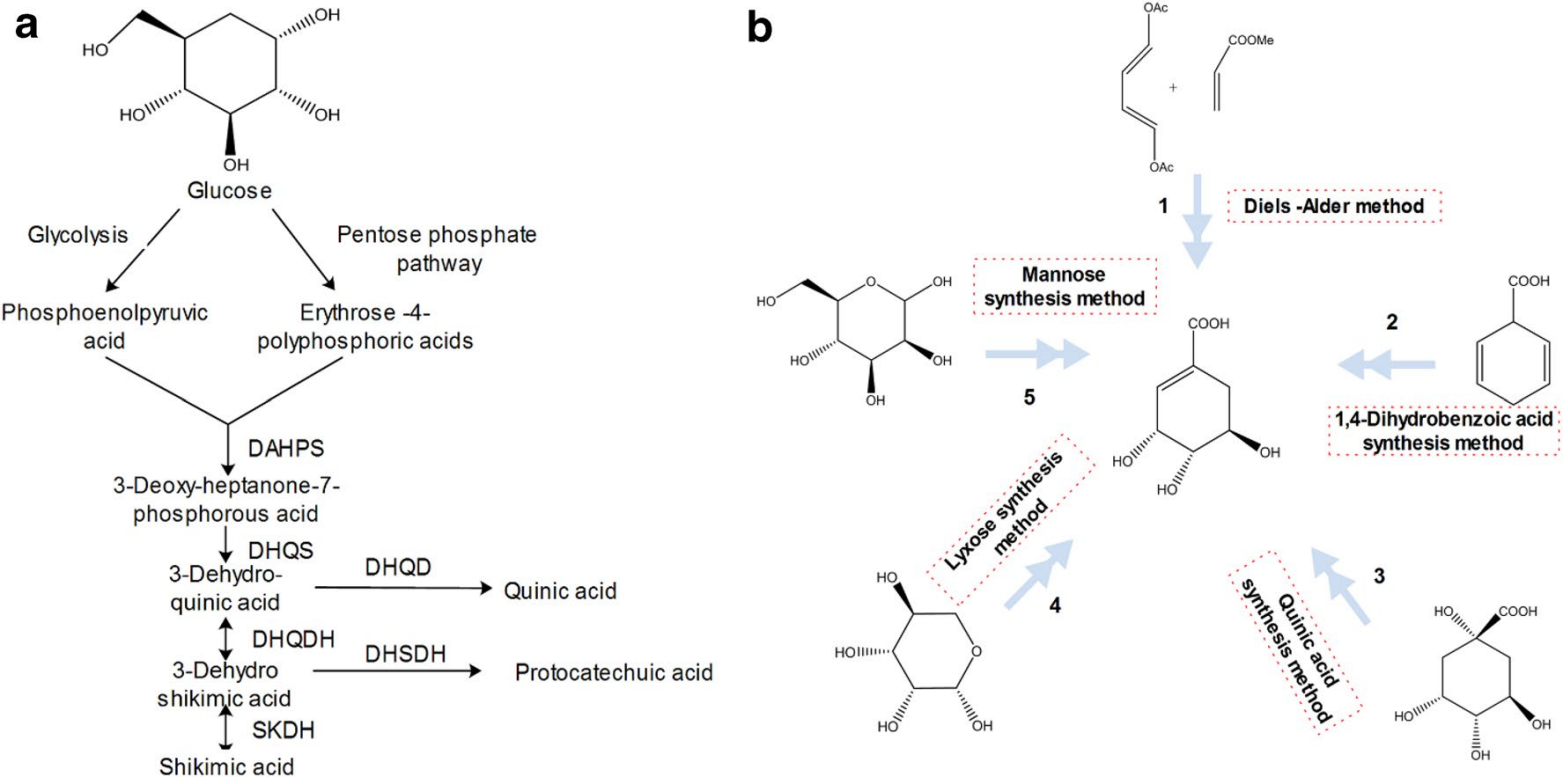
The biosynthesis pathways (**a**) and chemical synthesis pathways (**b**) of shikimic acid

As traditional herbal medicines, *Illicium* plants have been technically investigated on active ingredient synthesis. Here are two ways for shikimic acid synthesis including biosynthetic pathways and chemical pathways. Figure 1a shows the shikimate pathway, which serves as an intermediate product in this pathway to form essential aromatic amino acids [7–11]. The whole biosynthetic pathway of shikimic acid involves six steps with uncontrollable conditions and a low product yield of 36%. Besides, five routes of shikimic acid synthesis (Fig. 1b) have been reported [12–14]. Diels–Alder method [1, 9] possess a low product yield (15%). Quinic acid is used to produce shikimic acid with more than 7 days [8]. The route with the raw material of mannose which is a novel and simplified strategy has five steps and a low product yield of 25% [9]. Tamiflu plays a key role in resisting avian influenza [10, 15]. Tamiflu synthesis has been widely examined which need shikimic acid as raw material, and research on the suitable distribution areas of the 15 *Illicium* species has revealed that these plants as natural sources of shikimic acid can be efficient materials to obtain Tamiflu. Nevertheless, the product yield of shikimic acid is low and synthetic procedure is complex, and the possible ecologically suitable areas for *Illicium* plants are largely unknown [16, 17].

We collated high-quality ground-point data to analyze favorable growing areas for future applications, and we used seven environmental factors to understand the current climate conditions for *Illicium* plant farming. According to ecological niche, we can assess the habitats which are conducive for the growth of the 15 *Illicium* species by using a geographic information system for global medicinal plants (GMPGIS), a software could predict the potential distribution of species based on the climate factors analysis and has been applied in many species such as *Panax ginseng* [18]. Our analyses primarily focused on identifying their suitable habitats of *Illicium* species at a global scale and determining appropriate areas where they could be introduced and cultivated worldwide. This study could enhance our understanding of the distribution range of *Illicium* plants, address problems related to shikimic acid supply, and provide strategies to help protect *I. difengpi* B. N. Chamg, which is an endangered species.

## Methods

### Data sources of target species

*Illicium* plants are evergreen trees or shrubs, and they are suited to mountain climate. In this research, the occurrence data points of modeling species distribution were carefully screened to reduce sampling bias and sampling errors from four resources [7, 19]: (1) Chinese Virtual Herbarium (CVH) (http://www.cvh.ac.cn/); (2) National Specimen Information Infrastructure (NSII) (http://www.nsii.org.cn/2017/home.php); (3) literatures; and (4) field work. The number of sampling points is listed in Table 1. Using the website http://www.gpsspg.com/maps.htm, we can obtain the latitude and longitude of each point, and the range of the latitude and longitude of the sampling points is shown in Additional file 1: Table S1.

### Environmental variables for modeling

Environmental variables play important roles in determining the potential species’ distribution at a global scale. Hence, these variables are widely used to predict the possible suitable habitats of species. In the present study, the environmental characteristics of 15 *Illicium* species were characterized by determining seven environmental variables (annual mean temperature, mean temperature of the coldest quarter, annual precipitation, mean temperature of the warmest quarter, humidity, annual radiation, and soil), which were examined according to statistics, ecology, and botany, as well as their close relationship with plant growth.

### GMPGIS modeling

We modeled the potential regions of the 15 *Illicium* species by using the GMPGIS. This system has been verified successfully and has been used to predict potential distributions of many plant species, such as *Panax ginseng* C.A. Mey and *Panax notoginseng* (Burk.) F. H. et al. in China [18, 20, 21]. The following equations were utilized during the prediction of suitable distribution of the 15 *Illicium* species.

Data were standardized as follows:

$$ \chi^{\prime} = \frac{\chi - \hbox{min} }{\hbox{max} - \hbox{min} } $$

Similarity clustering analysis was conducted using the following equation:

$$ {\text{d}}_{\text{ij}} = \sqrt {(\chi_{11} - \chi_{12} )^{2} + (\chi_{12} - \chi_{22} )^{2} + {\text{L}} + (\chi_{{{\text{p}}1}} - \chi_{{{\text{p}}2}} )^{2} } = \left[ {\sum {\frac{\text{p}}{{{\text{k}} = 1}}(\chi_{\text{ki}} - \chi_{\text{kj}} )^{2} } } \right]^{{\frac{1}{2}}} $$

According to the results of distance calculation [*Mind*_*ij*_*, Maxd*_*ij*_], we reclassified the distance raster, and the areas at 99.9% similarity were considered the suitable distribution with maximum similarities. Besides, after the intersection between the distance layer which is reclassified and the soil layer, the coincident suitable areas for climate and soil conditions could be obtained.

The Minimum Standards of Reporting Checklist contains details of the experimental design, and statistics, and resources used in this study (Additional file 2).

## Results

### Analysis of seven environmental variables

**Fig. 2:**
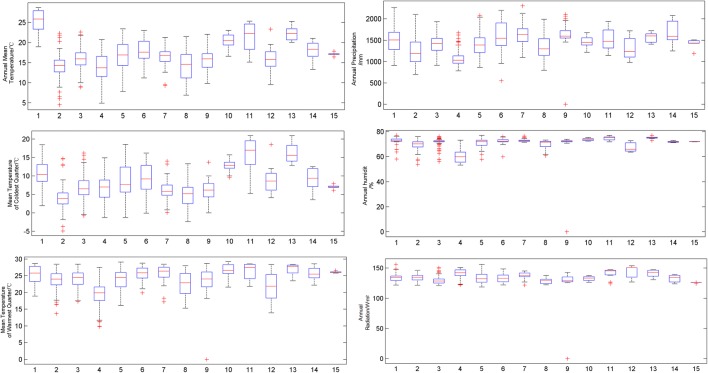
Boxplot chart of the environmental variables. (1, *I. verum*; 2, *I. henryi;* 3, *I. majus*; 4, *I. simonsii*; 5, *I. micranthum*; 6, *I. dunnianum*; 7, *I. lanceolatum*; 8, *I. fargesii*; 9, *I. jiadifengpi*; 10, *I. difengpi;* 11, *I. ternstroemioides*; 12, *I. macranthum; 13, I. oligandrum;* 14, *I. brevistylum*; 15, *I. pachyphyllum*)

The specific ranges of the seven environmental variables of the 15 *Illicium* species are shown in Table 2. Different species exist in various soil types. The boxplots containing six environmental variables of the 15 *Illicium* species are illustrated in Fig. 2. Most of the variables showed large ranges in group 1 followed by 15, especially annual mean temperature, mean temperature of the coldest quarter, and annual precipitation. These significant differences could be attributed to different growth environments required by various species.

**Table 2:** Range values of the environmental variables for 15 kinds of *Illicium* plants

Chinese name of species	Annual mean temperature/°C	Mean temperature of coldest quarter/°C	Mean temperature of warmest quarter/°C	Annual precipitation/mm	Annual humidity/%	Annual radiation /W m^−2^
*I. verum*	11.7–24.0	1.9–18.4	18.9–28.7	909–2266	58.2–77.1	121.9–156.0
	*Soil types* lixisols, acrisols, fluvisols, etc.					
*I. henryi*	4.5–22.3	− 4.9–14.7	13.7–28.5	697–2107	53.9–76.2	121.5–146.5
	*Soil types* lixisols, chernozems, plinthosols, arenosols, acrisols, ferralsols, leptosols, etc.					
*I. majus*	8.9–22.6	− 0.8–16.2	17.3–28.5	915–1933	56.2–76.1	120.7–150.6
	*Soil types* arenosols, lixisols, acrisols, rock debris, chernozems, inland waterways, etc.					
*I. simonsii*	4.9–20.7	− 1.3–14.9	9.8–27.5	782–1675	53.3–73.1	121.9–150.8
	*Soil types* chernozems, arenosols, acrisols, leptosols, lixisols, luvisols, etc.					
*I. micranthum*	7.8–23.4	− 1.3–18.5	16.1–29.1	865–2077	57.8–77.1	118.5–156.1
	*Soil types* acrisols, lixisols, arenosols, rhogosols, chernozems, fluvisols, etc.					
*I. dunnianum*	11.2–23.0	− 0.1–16.2	19.9–28.8	553–2203	59.9–76.0	122.1–148.6
	*Soil types* acrisols, lixisols, arenosols, chernozems, luvisols, rhogosols, etc.					
*I. lanceolatum*	9.3–21.2	0.1–14.0	17.2–28.5	1092–2305	71.4–76.3	121.8–145.1
	*Soil types* acrisols, lixisols, anthrosols, arenosols, chernozems, luvisols, etc.					
*I. fargesii*	6.9–21.5	− 2.4–13.3	15.3–28.0	793–1988	60.9–73.2	122.2–137.7
	*Soil types* acrisols, lixisols, arenosols, rhogosols, chernozems, etc.					
*I. jiadifengpi*	9.8–22.0	0.6–13.7	18.2–28.7	1457–2104	70.8–73.9	126–142.7
	*Soil types* acrisols, lixisols, arenosols, rhogosols, etc.					
*I. difengpi*	16.6–23.0	9.6–15.7	21.6–29.2	1216–1680	72.4–75.2	126.4–137.7
	*Soil types* acrisols, lixisols, arenosols, rhogosols, chernozems, leptosols, etc.					
*I. ternstroemioides*	15.1–25.3	5.2–20.9	21.8–28.6	1143–1941	71.8–76.9	123.9–147.6
	*Soil types* acrisols, fluvisols, chernozems, chernozems, etc.					
*I. macranthum*	9.5–23.3	4.1–18.5	13.9–28.4	977–1719	63.5–72.7	127.0–153.8
	*Soil types* acrisols, lixisols leptosols, etc.					
*I. oligandrum*	20.0–25.2	12.8–20.9	23.5–28.4	1408–1722	72.9–76.9	130.6–147.6
	*Soil types* acrisols, fluvisols, lixisols, arenosols, etc.					
*I. brevistylum*	13.3–21.0	3.5–12.5	22.2–28.6	1247–2077	71.1–72.9	123.9–139.4
	*Soil types* acrisols, chernozems, etc.					
*I. pachyphyllum*	16.4–17.8	6.1–7.9	25.8–26.6	1190–1505	71.9–72.1	124.1–126.5
	*Soil types* acrisols, etc.

### Ecological niche

**Fig. 3:**
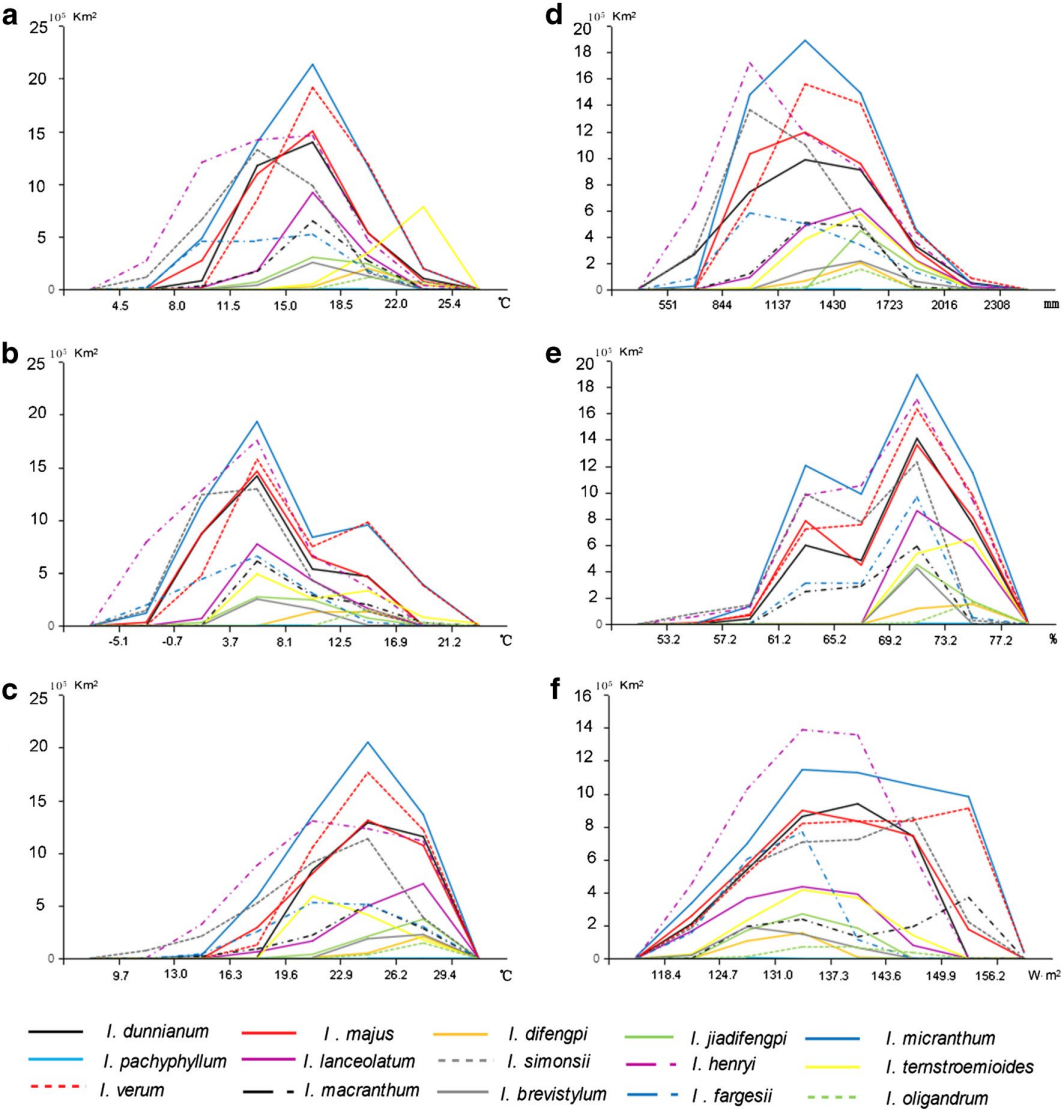
The ecological niche of every environmental variables. **a** Annual mean temperature; **b** mean temperature of coldest quarter; **c** mean temperature of warmest quarter; **d** annual precipitation; **e** annual humidity and annual radiation

Each environmental variable’ range was divided into six segments on average, and the area (km^2^) of each segment for the 15 *Illicium* species is shown in Fig. 3. These plants were widely distributed at annual mean temperature of 11.5 –22.0 °C, mean temperature of − 0.7–16.9 °C in the coldest quarter, and annual precipitation of 551–2016 mm. Furthermore, the potential suitable areas were larger at a mean temperature of 19.6–29.4 °C in the warmest quarter, humidity of 61.2–75.2%, and annual radiation of 124.7–156.2 W m^2^.

### Analysis of the suitable distribution of the 15 *Illicium* species

**Fig. 4:**
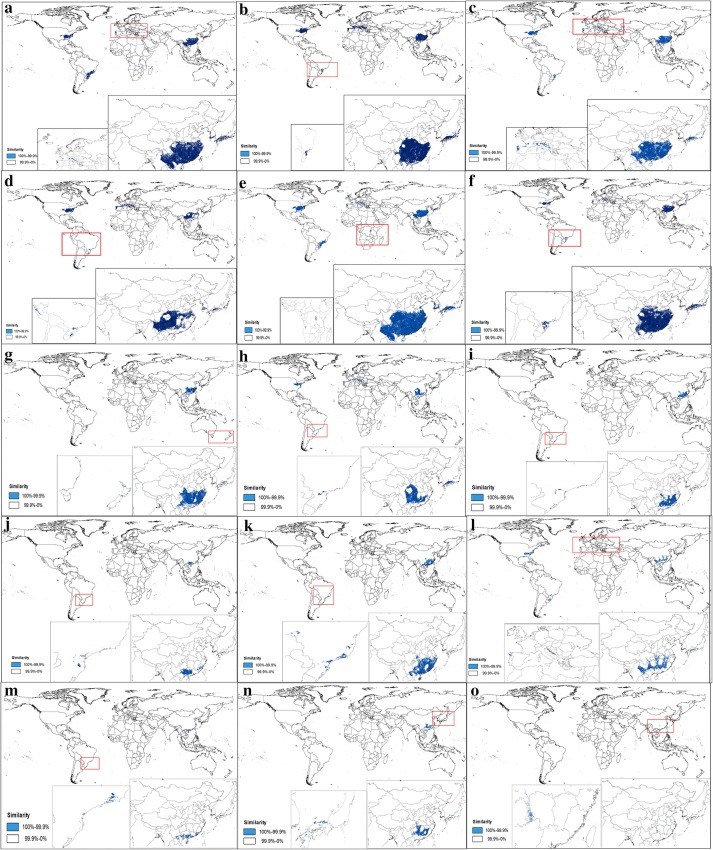
Suitable areas in China and in the world of 15 *illicium* plants. (**a** *I. verum*; **b** *I. henryi;* **c** *I. majus*; **d** *I. simonsii*; **e** *I. micranthum*; **f** *I. dunnianum;* **g** *I. lanceolatum*; **h** *I. fargesii*; **i** *I. jiadifengpi*; **j** *I. difengpi;* **k** *I. ternstroemioides*; **l** *I. macranthum;* **m** *I. oligandrum;* **n** *I. brevistylum*; **o** *I. pachyphyllum*)

**Fig. 5:**
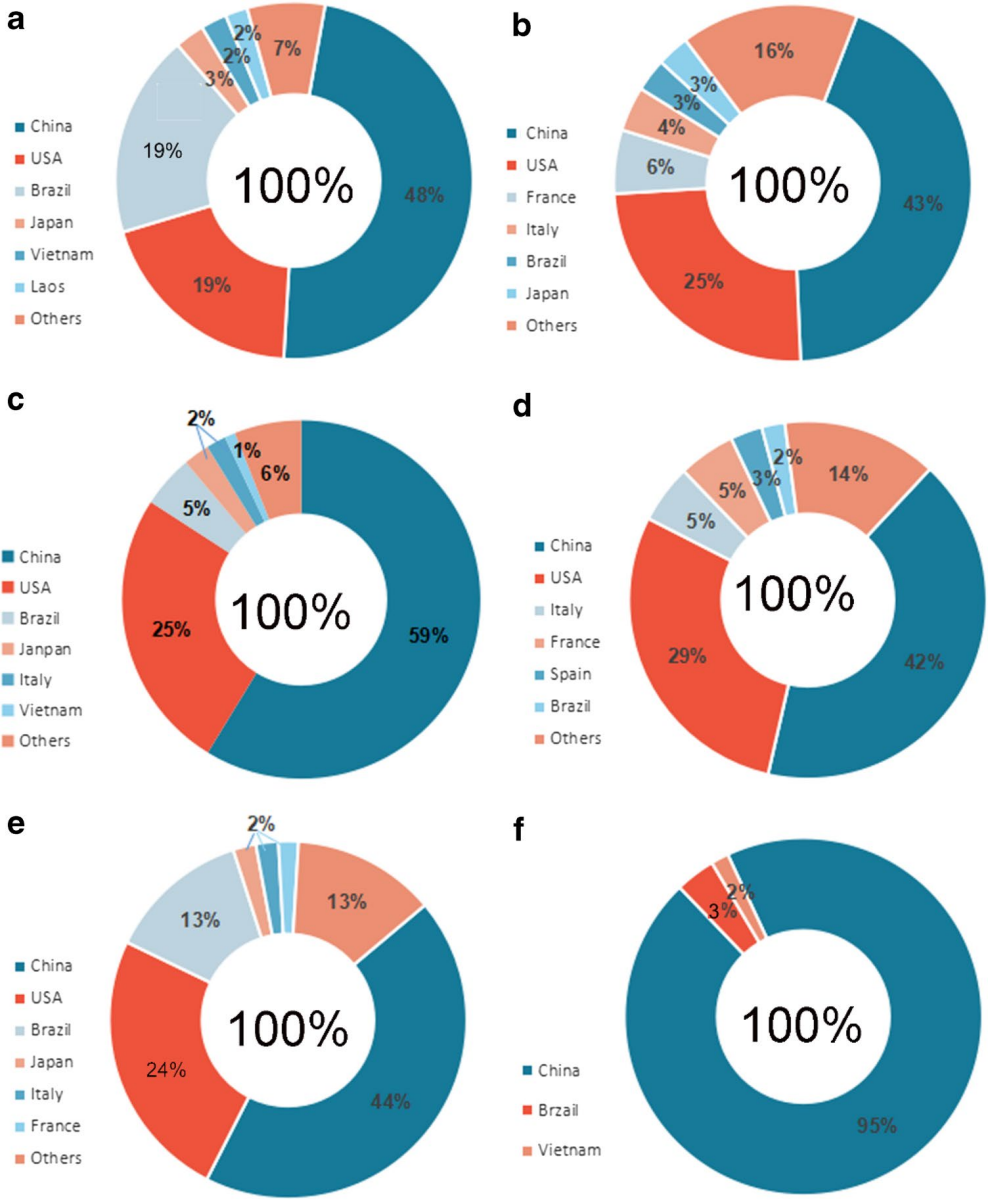
The ratio of suitable areas in the world of six typical *Illicium* plants. (**a** *I. verum;* **b** *I. henryi*; **c** *I. majus*; **d** *I. simonsii*; **e** *I. micranthum,* **f** *I. difengpi*)

The suitable habitat maps based on the occurrence points of the 15 *Illicium* species in CVH, NSII databases, and field work are shown in Figs. 4 and 5 and in Additional file 3: Figure S1. The 15 *Illicium* species are mainly located in the latitude range of 45°S–45°N, which is south and southeast of China, east of the USA, and south of Brazil. The core area in China where these plants are mainly distributed is 1357.68 × 10^4^ km^2^, which ranks first and accounts for 56% of the total area worldwide. This ranking is followed by the USA with suitable areas of 527.42 × 10^4^ km^2^. Figure 5 demonstrates that most of the areas in China are climatically suitable for *I. difengpi*, and only a small area spanning approximately 5% exists outside of China. Additional file 3: Figure S1 shows that *I. pachyphyllum*is located only in China.

**Fig. 6:**
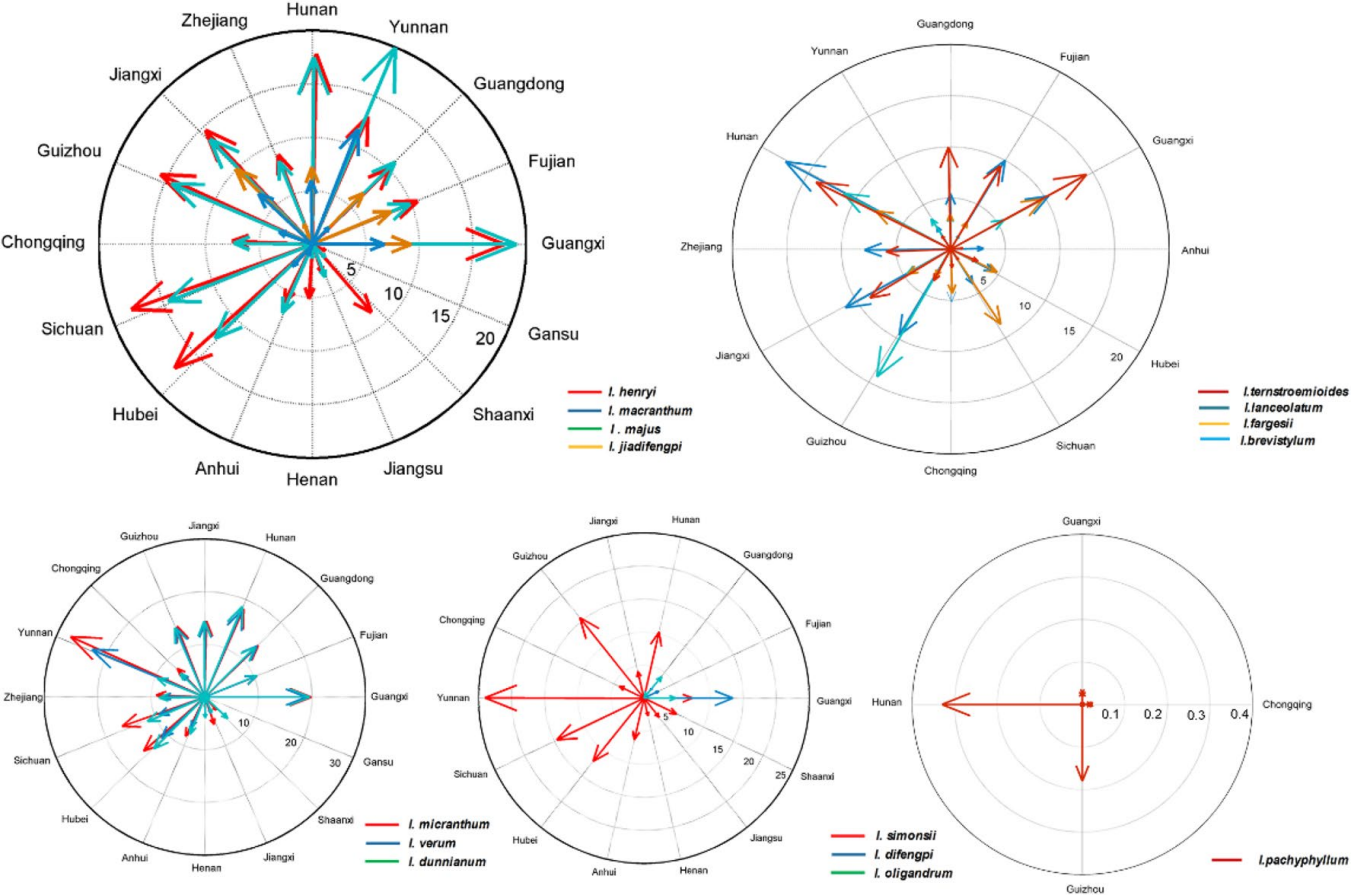
Suitable areas in every provinces in China of 15 *illicium* plants

According to the map, the potential suitable habitats of the 15 *Illicium* species are found in six provinces in China (Fig. 6): Guangxi, Yunnan, Hunan, Guizhou, Sichuan, and Hubei. The specific suitable areas of each *Illicium* plant in China and other countries are shown in Additional file 1: Tables S2, S3.

- *I. verum* Hook. f, an edible plant whose main producing area is Guangxi from China, which is recognized by National Health and Family Planning Commission, has a potential habitat with a total area of 377.9 × 10^4^ km^2^ (19%) worldwide. It also covers approximately 64.04 × 10^4^ km^2^ (18%) in the USA and 60.5 × 10^4^ km^2^ (48%) in Brazil. The core areas are approximately 158.8 × 10^4^ km^2^, which are mainly located in Yunnan, Guangxi, Hunan, and other places in China.
- *I. henryi* Diels is a Chinese endemic species and its main producing areas are Shannxi and Gansu. Its favorable area measured approximately 407.4 × 10^4^ km^2^ worldwide. It is distributed at 100.3 × 10^4^ km^2^ (25%) in the USA, which has the second-largest coverage. This species also thrives at 23.3 × 10^4^ (6%) and 16.4 × 10^4^ km^2^ (4%) in France and Italy, respectively. The core areas cover 176.3 × 10^4^ km^2^ (43%) in Yunnan, Guangxi, Guangdong, and Hunan in China.
- *I. majus* Hook. f is mainly produced in Hunan which has a suitable habitat with an area of 275.9 × 10^4^ km^2^ worldwide. Its favorable habitats in the USA, Brazil, and Japan cover 70.1 × 10^4^ (25%), 13.1 × 10^4^ (5%), and 6.5 × 10^4^ km^2^ (6%), respectively. The core areas account for 59%, or approximately 62.0 × 10^4^ km^2^, of the land area in Guangxi, Yunnan, Hunan, and Sichuan in China.
- *I. simonsii* Maxim is prevailingly occurred in Guizhou and Sichuan. It has a globally potential suitable area of 272.6 × 10^4^ km^2^. It covers 78.9 × 10^4^ (29%), 14.5 × 10^4^ (5%), 14.0 × 10^4^ (5%), and 5.7 × 10^4^ km^2^ (3%) suitable areas in the USA, Spain, Italy, and France, respectively. The core areas spanning 113.5 × 10^4^ km^2^ (42%) are mainly distributed in Yunnan, Hunan, and Guizhou provinces in China.
- *I. micranthum* Dunn, is a native species chiefly in Fujian and Hubei, in China. The area of its suitable habitat is approximately 435.37 × 10^4^ km^2^. Its distribution areas in the USA, Brazil, and Japan are 107.86 × 10^4^ (24%), 56.38 × 10^4^ (13%), and 9.04 × 10^4^ km^2^ (13%), respectively. The core areas spanning 190.7 × 10^4^ km^2^ (44%) are mainly located in Hunan, Guangxi, and Yunnan in China.
- *I. dunnianum* Tutch, is a species endemic to China mainly in Fujian and Hunan. Its suitable habitat worldwide has a land area of 263.1 × 10^4^ km^2^. This species also covers an area of 47.2 × 10^4^ km^2^ (18%) in the USA. The core areas spanning 62% are mainly distributed in China, especially in Guangxi, Hunan, and Jiangxi provinces.
- *I. lanceolatum* A. C. Smith is a native species in China and mostly in Jiangsu and Anhui. It possess a potential suitable area of 109.1 × 10^4^ km^2^ worldwide. This species covers an area of approximately 8% of the total distribution area in Brazil. It is also distributed in Vietnam, Japan, and the USA with respective areas of 1.4 × 10^4^, 1.6 × 10^4^, and 0.2 × 10^4^ km^2^, which account for 1%. Its potential suitable habitat with an area of 95.1 × 10^4^ km^2^ (87%) is located in China, particularly in Hunan, Jiangxi, and Guangxi provinces.
- *I. fargesii* Finet & Gagnep is a species endemic to China, its main producing areas are Guizhou and Hunan. It has a suitable area of 135.2 × 10^4^ km^2^. This species covers 31.8 × 10^4^ (24%) and 5.8 × 10^4^ km^2^ (4%) in the USA and Japan. Its potential distribution area in China is 71.7 × 10^4^ km^2^ (53%) spanning Guizhou, Hunan, and Guangxi provinces.
- *I. jiadifengpi* B. N. Chang principally grows in Anhui and Zhejiang, which has a suitable habitat with an area of 45.4 × 10^4^ km^2^ worldwide. This species respectively covers 0.6 × 10^4^ and 0.5 × 10^4^ km^2^, or equivalent to 1% of the total area worldwide, in Japan and Brazil. A large proportion of the area corresponding to 45.4 × 10^4^ km^2^ (98%) is mainly distributed in Anhui, Zhejiang, Guangxi, and Guangdong provinces in China.
- *I. difengpi* B. N. Chamg prevailingly grows in Guangxi and Yunnan, China and is recorded as endangered in the Endangered Species Act, it covers a potential suitable area of 20.1 × 10^4^ km^2^. This species is distributed in 0.7 × 10^4^ (3%) and 0.3 × 10^4^ km^2^ (2%) in Vietnam and Brazil, respectively. Its largest suitable area is 19.0 × 10^4^ km^2^ (95%), which is mainly located in Guangxi and southeast of Yunnan in China.
- *I. ternstroemioides* A. C. Smith, a native species in China, mainly in Hainan, has a total potential suitable area of 88.7 × 10^4^ km^2^. This species covers 6.8 × 10^4^ (8%) and 4.3 × 10^4^ km^2^ (5%) in Brazil and Vietnam, respectively. The core areas measuring 76.7 × 10^4^ km^2^ (85%) are mainly located in Hainan, Fujian, Hunan, and Guangdong provinces in China.
- *I. macranthum* A. C. Smith primarily occurred in Yunnan, China, has a total potential suitable area of 89.9 × 10^4^ km^2^. This species is distributed in areas of 27.3 × 10^4^ (30%) and 9.6 × 10^4^ km^2^ (12%) in the USA and Brazil, respectively. This species also occupies the largest suitable habitat area of 44.6 × 10^4^ km^2^ (49%) found in Yunnan and Jiangxi provinces in China.
- *I. oligandrum* Merr.et Chun, chiefly grows in Guangxi and Hainan from China, has a total potential suitable area of 13.6 × 10^4^ km^2^. This species respectively covers 2.53 × 10^4^ (18%) and 9.9 × 10^4^ km^2^ in Vietnam and China (Guangdong and Guangxi provinces), and this coverage accounts for 73% of the total area worldwide.
- *I. brevistylum* A. C. Smith, a species endemic to China, mainly in Fujian and Hunan, has a globally potential distribution area of 31.8 × 10^4^ km^2^. It covers 0.6 × 10^4^ km^2^ (2%) in Japan and 31.2 × 10^4^ km^2^ (98%) in other regions, such as Fujian, Guangdong, Guangxi, and Hunan provinces in China.
- *I. pachyphyllum* A. C. Smith, another species endemic to China, principally in Hunan and Guangdong, has a potential suitable habitat with an area of 0.5 × 10^4^ km^2^ in Hunan, Guizhou, and Guangxi provinces in China. This observation is consistent with our current findings.

## Discussion

In this study, spatial analysis was conducted using GMPGIS to define the spatial distribution of habitat suitability and the range of each variable for two classes of habitats of 15 *Illicium* species. According to the analysis of environmental variables, it is easy to be concluded that different *Illicium* species exist in different requirement on a growing environment. In addition, Our GMPGIS models successfully showed that the most suitable climate condition for these plants are annual mean temperature of 11.5–22.0 °C, mean temperature of − 0.7–16.9 °C in the coldest quarter, and annual precipitation of 551–2016 mm, in which are roughly consistent with the known regions where the 15 *Illicium* species exist and mostly located in a large range from S 45° to N 45°. On the basis of the climatic suitable maps of these plants, we can conclude that they covered an area of 1356.75 × 10^4^ km^2^ in China, which ranked first, followed by the USA with an area of 527.42 × 10^4^ km^2^. We speculated that the reason for China topped the rankings is not only China has vast areas could grow *Illicium* plants, but also there are many endemic species in China such as *I. fargesii*, *I. brevistylum* and *I. pachyphyllum*. *I. verum* with a high shikimic acid content is mainly distributed in China (48%) and in the USA (19%). The suitable habitats of *I. difengpi* cover 95, 3, and 2% in China, Brazil, and Vietnam, respectively.

## Conclusion

According to the data on suitable habitats, we recommended that *I. verum* can be expanded to suitable regions, such as southeast of China and south of Brazil, to solve problems on shikimic acid shortage. *I. difengpi* can be introduced to Vietnam and Brazil and can thus mitigate its risk of becoming endangered. Some species, such as *I. henryi*, *I. micranthum*, and *I. dunnianum*, which are endemic to China, can be introduced and cultivated in the USA or other favorable regions. This approach possibly addresses the high demands for shikimic acid, protects the diversity of rare or endangered species, and helps governments establish various protection strategies for future ecological conservation.

## Additional files

**Additional file 1: Table S1.** The range of the latitude and longitude of the sampling points. **Table S2.** The specific areas in each countries of the world. **Table S3.** The specific areas in each provinces of China.

**Additional file 2.** Minimum standards of reporting checklist.

**Additional file 3: Figure S1.** The ratio of suitable areas in the world of the other 9 Illicium plants (a, *I. dunnianum*; b, *I. lanceolatum*; c, *I. fargesii*; d, *I. jiadifengpi*; e, *I. ternstroemioides*; f, *I. macranthum;* g*, I. oligandrum*; h,*I. brevistylum*; i, *I. pachyphyllum*).

## Abbreviations

GMPGIS: geographic information system for global medicinal plants
CVH: Chinese Virtual Herbarium
NSII: National Specimen Information Infrastructure
