# Supplementary material for: Global ecological regionalization of 15 Illicium species: nature sources of shikimic acid
Source: Chin Med. 2018 Jun 15;13:31. doi: 10.1186/s13020-018-0186-9 (PMC6003141; doi:10.1186/s13020-018-0186-9)
Supplement: Supplementary file 1 — Additional file 1: Table S1. The range of the latitude and longitude of the sampling points. Table S2. The specific areas in each countries of the world. Table S3. The specific areas in each provinces of China. [file 13020_2018_186_MOESM1_ESM.docx]

**Additional file 1**

**Table S1 The range of** **the latitude and longitude of the sampling points**

| **Latin names  of species** | **longitude** | **latitude** |
| --- | --- | --- |
| *I. verum* | 95.3319422713 -115.0816566451 | 18.9808530747 -32.7303937835 |
| *I. henryi* | 101.9174772276 -119.7526288033 | 23.1781544870 -33.6355078958 |
| *I . majus* | 100.7908152308 -119.6813038047 | 22.6046101244 -31.8056230450 |
| *I. simonsii* | 98.4114937051 -114.7937423415 | 22.994418172 -31.7596910903 |
| *I. micranthum* | 99.3755491729 -116.2846016407 | 18.8984090879 -32.4635684405 |
| *I. dunnianum* | 105.1687651992 -118.3037816332 | 21.7199746909 -35.0493015974 |
| *I. lanceolatum* | 106.5754079819 -120.5150842667 | 23.7263868242 -30.5931767980 |
| *I . fargesii* | 103.1765556335 -117.8615203856 | 23.5240113081 -32.0507080935 |
| *I. jiadifengpi* | 108.1047713757 -119.2098999023 | 23.9331424571 -29.2469834336 |
| *I. difengpi* | 104.6693015099 -109.8949098587 | 22.3433181736 -25.1083730569 |
| *I. ternstroemioides* | 103.5582232475 -117.8231978416 | 18.2084801960 -30.6919780000 |
| *I. macranthum* | 100.5628180504 -114.0293552092 | 22.0071342669 -25.8556127014 |
| *I. oligandrum* | 107.8516674042 -116.7038798332 | 18.5600182669 -23.9012384122 |
| *I. brevistylum* | 108.2691584610 -114.1453002689 | 23.6376102956 -26.4600314102 |
| *I. pachyphyllum* | 108.7796258926 -109.9018621445 | 25.8906642103 -27.8545769240 |

| Countries | **Suitable areas（10^4^ Km^2^）** | | | | | | | | | | | | | | |
| --- | --- | --- | --- | --- | --- | --- | --- | --- | --- | --- | --- | --- | --- | --- | --- |
|  | *I. verum* | *I. henryi* | *I . majus* | *I. simonsii* | *I. micranthum* | *I. dunnianum* | *I. lanceolatum* | *I . fargesii* | *I. jiadifengpi* | *I. difengpi* | *I. ternstroemioides* | *I. macranthum* | *I. oligandrum* | *I. brevistylum* | *I. pachyphyllum* |
| China | 158.78 | 176.26 | 162.04 | 113.49 | 190.74 | 162.57 | 95.07 | 72.28 | 45.41 | 19.02 | 75.72 | 44.36 | 9.88 | 31.20 | 0.53 |
| USA | 64.04 | 100.28 | 70.14 | 78.91 | 107.86 | 47.19 | 0.17 | 31.80 | - | - | - | 27.03 | - | - | - |
| Brazil | 60.53 | 12.06 | 13.05 | 5.72 | 56.38 | 11.18 | 8.36 | 1.37 | 0.47 | 0.73 | 6.84 | 9.63 | 0.70 | - | - |
| Japan | 8.88 | 11.91 | 6.50 | 3.69 | 9.04 | 9.57 | 1.62 | 5.82 | 0.60 | - | - | 1.29 | - | 0.57 | - |
| Vietnam | 7.78 | 2.26 | 2.65 | 0.06 | 7.09 | 2.71 | 1.41 | - | 0.01 | 0.32 | 4.27 | - | 2.53 | - | - |
| Laos | 6.65 | 0.07 | 2.33 | 0.07 | 6.69 | 1.25 | - | - | - | - | 0.98 | 1.00 | 0.36 | - | - |
| [Myanmar](C://Users/Administrator/AppData/Local/youdao/dict/Application/7.0.1.0227/resultui/dict/?keyword=Myanmar) | 4.14 | - | 0.29 | 0.96 | 3.64 | 0.06 | - | - | - | - | - | 0.69 | - | - | - |
| Italy | 3.65 | 16.40 | 4.89 | 14.45 | 8.85 | 12.79 | 0.02 | 3.14 | - | - | - | 0.06 | - | - | - |
| [Portugal](C://Users/Administrator/AppData/Local/youdao/dict/Application/7.0.1.0227/resultui/dict/?keyword=Portugal) | 2.28 | 4.67 | 3.11 | 4.44 | - | 3.66 | 0.17 | 2.39 | - | - | - | 0.14 | - | - | - |
| [Korea](C://Users/Administrator/AppData/Local/youdao/dict/Application/7.0.1.0227/resultui/dict/?keyword=Korea) | 1.32 | - | 0.20 | 0.83 | 2.79 | 0.07 | - | - | - | - | - | 0.05 | - | - | - |
| [Canada](C://Users/Administrator/AppData/Local/youdao/dict/Application/7.0.1.0227/resultui/dict/?keyword=Canada) | - | 2.35 | - | - | 0.06 | - | - | - | - | - | - | - | - | - | - |
| [France](C://Users/Administrator/AppData/Local/youdao/dict/Application/7.0.1.0227/resultui/dict/?keyword=France) | 1.22 | 23.36 | 2.96 | 14.02 | 7.88 | 2.12 | 0.03 | 7.41 | - | - | - | 0.17 | - | - | - |
| Spain | 1.03 | 8.81 | 2.41 | 7.87 | 3.58 | 1.06 | - | 2.46 | - | - | - | 0.88 | - | - | - |
| [Croatia](C://Users/Administrator/AppData/Local/youdao/dict/Application/7.0.1.0227/resultui/dict/?keyword=Croatia) | 1.02 | 4.38 | 1.28 | 3.38 | - | - | - | - | - | - | - | - | - | - | - |
| [India](C://Users/Administrator/AppData/Local/youdao/dict/Application/7.0.1.0227/resultui/dict/?keyword=India) | 0.23 | - | - | 0.03 | 0.36 | - | - | - | - | - | - | - | - | - | - |
| [Mexico](C://Users/Administrator/AppData/Local/youdao/dict/Application/7.0.1.0227/resultui/dict/?keyword=Mexico) | 0.31 | - | - | - | 0.26 | - | - | - | - | - | 0.02 | 0.05 | - | - | - |
| [Australia](C://Users/Administrator/AppData/Local/youdao/dict/Application/7.0.1.0227/resultui/dict/?keyword=Australia) | 0.02 | 4.64 | 0.37 | - | 1.16 | - | - | 0.12 | - | - | - | 0.53 | - | - | - |
| Thailand | - | - | - | - | - | - | - | - | - | - | - | - | - | - | - |
| [South](C://Users/Administrator/AppData/Local/youdao/dict/Application/7.0.1.0227/resultui/dict/?keyword=South) [Africa](C://Users/Administrator/AppData/Local/youdao/dict/Application/7.0.1.0227/resultui/dict/?keyword=Africa) | 0.15 | - | - | - | - | - | - | - | - | - | - | - | - | - | - |
| [Tanzania](C://Users/Administrator/AppData/Local/youdao/dict/Application/7.0.1.0227/resultui/dict/?keyword=Tanzania) | - | - | - | - | 0.23 | - | - | - | - | - | - | 0.04 | - | - | - |
| Turkey | 0.58 | 0.23 | 0.18 | - | 1.01 | 0.61 | - | - | - | - | - | 0.16 | - | - | - |

**Table S2 The specific areas in each countries of the world**

**Table S3 The specific areas in each provinces of China**

| **Provinces** | **Suitable areas（10^4^ Km^2^）** | | | | | | | | | | | | | | |
| --- | --- | --- | --- | --- | --- | --- | --- | --- | --- | --- | --- | --- | --- | --- | --- |
|  | *I. verum* | *I. henryi* | *I . majus* | *I. simonsii* | *I. micranthum* | *I. dunnianum* | *I. lanceolatum* | *I . fargesii* | *I. jiadifengpi* | *I. difengpi* | *I. ternstroemioides* | *I. macranthum* | *I. oligandrum* | *I. brevistylum* | *I. pachyphyllum* |
| Guangxi | 18.93 | 17.96 | 19.08 | 7.33 | 19.87 | 19.63 | 10.35 | 9.79 | 9.24 | 13.43 | 14.41 | 6.74 | 4.81 | 5.65 | 0.03 |
| Fujian | 10.43 | 10.63 | 9.87 | 0.32 | 10.46 | 10.56 | 10.01 | 3.07 | 7.94 | 2.48 | 9.34 | 0.21 | 0.48 | 1.53 | — |
| Guangdong | 13.70 | 10.13 | 10.87 | 1.81 | 13.92 | 13.30 | 5.44 | 3.35 | 6.72 | 2.15 | 9.96 | 2.17 | 4.21 | 3.44 | — |
| Yunnan | 22.87 | 12.98 | 19.93 | 24.01 | 27.49 | 9.47 | 2.53 | 3.47 | 0.05 | 0.71 | 1.53 | 11.57 | 0.18 | — | — |
| Hunan | 17.80 | 18.22 | 17.48 | 10.26 | 18.26 | 18.60 | 17.37 | 11.23 | 7.19 | 0.03 | 13.95 | 5.95 | 0.97 | 7.80 | 0.33 |
| Zhejiang | 7.91 | 8.97 | 8.37 | 0.09 | 9.06 | 8.55 | 7.91 | — | 2.05 | — | 5.91 | — | — | — | — |
| Jiangxi | 14.13 | 14.62 | 13.71 | 4.16 | 14.44 | 14.41 | 11.21 | 4.72 | 10.05 | — | 8.79 | 6.91 | — | 4.60 | — |
| Guizhou | 14.14 | 15.64 | 14.33 | 15.58 | 14.37 | 14.30 | 9.62 | 14.18 | 0.04 | 0.23 | 3.45 | 3.34 | — | 2.97 | 0.18 |
| Chongqing | 5.97 | 7.52 | 7.37 | 4.16 | 7.44 | 6.40 | 5.17 | 4.32 | 0.25 | — | 1.82 | 0.60 | — | 1.34 | 0.02 |
| Sichuan | 9.21 | 18.02 | 14.52 | 14.61 | 16.35 | 11.62 | 3.94 | 8.65 | — | — | 1.26 | 0.80 | — | 0.22 | — |
| Hubei | 11.21 | 17.33 | 12.66 | 12.25 | 15.33 | 13.65 | 4.52 | 4.82 | 1.19 | — | 2.78 | 2.80 | — | 0.29 | — |
| Anhui | 6.23 | 6.05 | 7.01 | 6.50 | 8.28 | 7.55 | 3.01 | — | 0.67 | — | 1.13 | 1.11 | — | — | — |
| Henan | 1.10 | 5.12 | 1.31 | 2.74 | 1.84 | 3.96 | — | — | — | — | — | 0.02 | — | — | — |
| Jiangsu | 3.28 | 2.80 | 3.28 | 3.72 | 5.50 | 3.18 | 0.37 | — | — | — | — | 0.18 | — | — | — |
| Shaanxi | 0.87 | 8.40 | 1.71 | 5.47 | 3.27 | 5.90 | 0.01 | 4.01 | — | — | — | 0.04 | — | — | — |
| Shandong | — | — | — | — | 0.67 | — | — | — | — | — | — | — | — | — | — |
| Liaoning | — | — | — | — | — | — | — | — | — | — | — | — | — | — | — |
| Gansu | — | 1.31 | — | 0.16 | — | 0.94 | — | 0.10 | — | — | — | — | — | — | — |
| Shanghai | 0.42 | 0.55 | 0.43 | 0.19 | 0.54 | 0.42 | 0.08 | — | — | — | — | — | — | — | — |
| Shanxi | — | — | — | — | — | 0.04 | — | — | — | — | — | — | — | — | — |
| Xizang | 0.18 | — | 0.02 | 0.16 | 0.38 | — | — | — | — | — | — | — | — | — | — |
| Taiwan | 0.97 | 0.11 | 0.14 | — | 0.69 | 0.21 | 0.11 | — | — | — | 0.29 | — | 0.12 | — | — |
| Jilin | — | — | — | — | — | — | — | — | — | — | — | — | — | — | — |
| Hainan | 0.51 | 0.02 | 0.08 | — | 0.47 | 0.08 | — | — | — | — | 1.84 | — | 0.97 | — | — |
| Hongkong | 0.06 | — | — | — | 0.02 | 0.05 | — | — | — | — | 0.01 | — | — | — | — |
