# Supplementary material for: Global ecological regionalization of 15 Illicium species: nature sources of shikimic acid
Source: Chin Med. 2018 Jun 15;13:31. doi: 10.1186/s13020-018-0186-9 (PMC6003141; doi:10.1186/s13020-018-0186-9)
Supplement: Supplementary file 3 — Additional file 3: Figure S1. The ratio of suitable areas in the world of the other 9 Illicium plants (a, I. dunnianum; b, I. lanceolatum; c, I. fargesii; d, I. jiadifengpi; e, I. ternstroemioides; f, I. macranthum; g, I. oligandrum; h,I. brevistylum; i, I. pachyphyllum). [file 13020_2018_186_MOESM3_ESM.docx]

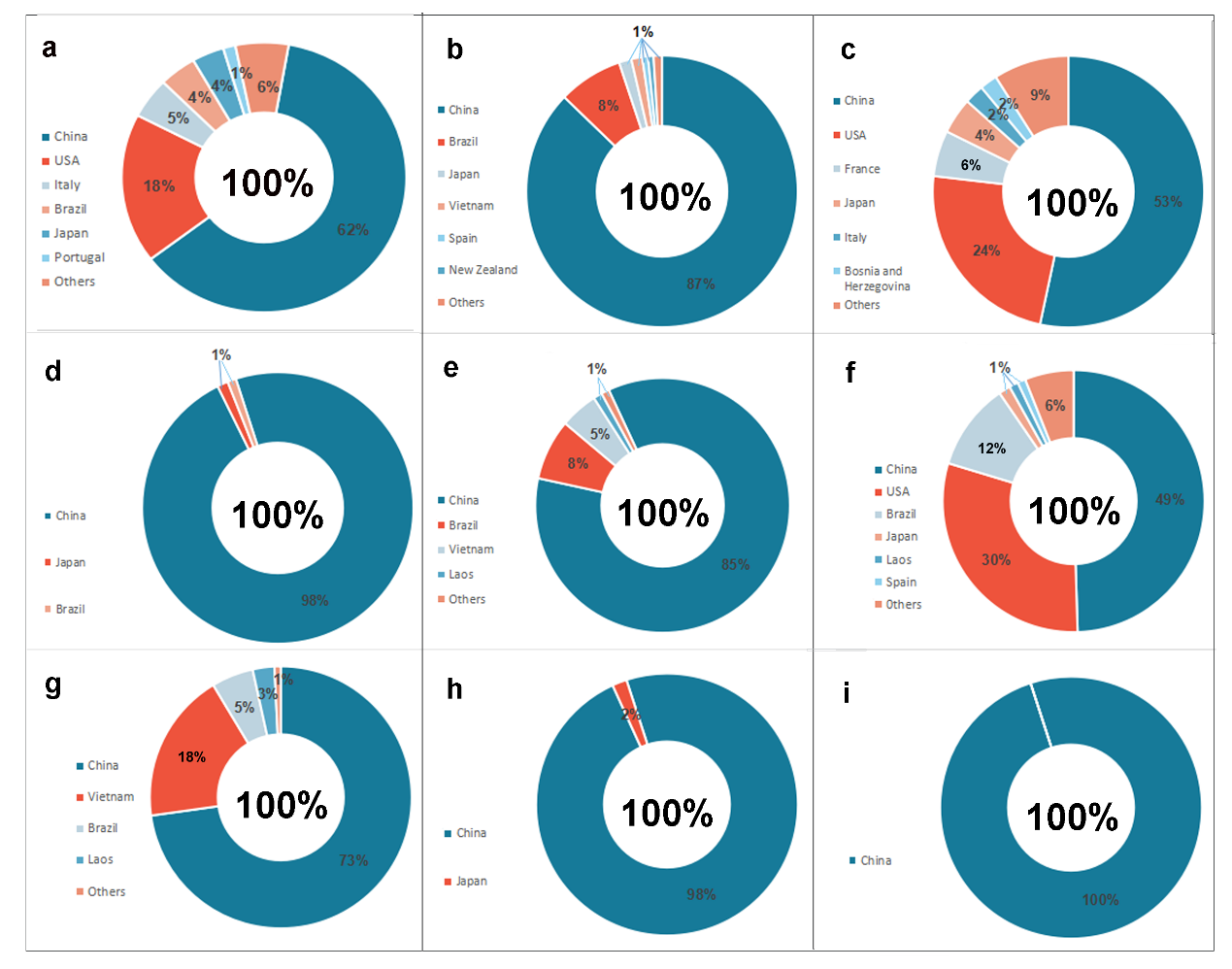


**Figure S1 The ratio of suitable areas in the world of the other 9 Illicium plants**

(a,*I. dunnianum* ; b,*I. lanceolatum*; c,h*I . fargesii*; d,*I. jiadifengpi*; e, *I. ternstroemioides*; f,*I. macranthum;* g*,I. oligandrum* ; h,*I. brevistylum*; i,*I. pachyphyllum*）
